# Facilitating translation of trial findings into NHS practice: case study of the Support and Treatment After Replacement (STAR) care pathway

**DOI:** 10.1302/2633-1462.66.BJO-2024-0043.R1

**Published:** 2025-06-05

**Authors:** Wendy Bertram, Vikki Wylde, Tom Woodward, Rachael Gooberman-Hill, Michael R Whitehouse, Nick Howells

**Affiliations:** 1 Bristol Medical School, University of Bristol, Bristol, UK; 2 National Institute for Health and Care Research Bristol Biomedical Research Centre, University Hospitals Bristol and Weston NHS Foundation Trust and the University of Bristol, Bristol, UK; 3 North Bristol NHS Trust, Bristol, UK

**Keywords:** Complex intervention, Total knee arthroplasty, Behaviour change, Translation, total knee arthroplasty (TKA), clinicians, chronic pain, orthopaedic surgeons, COVID-19 pandemic, physiotherapists, knee surgeons, knee arthroplasties, patient-reported outcome measures (PROMs), RCT

## Abstract

**Aims:**

The Support and Treatment After Replacement (STAR) care pathway is a clinically important, cost-effective treatment to improve pain outcomes over one year for people with chronic pain three months after total knee arthroplasty. This work describes the implementation of trial findings in practice at one NHS hospital and the further work undertaken to support national implementation.

**Methods:**

Trial findings were presented to NHS managers with a business case for a pilot embedded in usual care. Trial documentation was adapted using the capability, opportunity, motivation, and behaviour (COM-B) model for behaviour change and evidence-based approaches to increase questionnaire responses. Trial sites were contacted to understand their capacity to pilot the intervention.

**Results:**

The STAR care pathway was successfully implemented into NHS practice with a screening response rate of 89%. It is now permanently part of usual care at North Bristol NHS Trust. Trial centres indicated that lack of trained extended scope practitioners was a barrier to implementation. The trial manual and training sessions were adapted into an online training package.

**Conclusion:**

The STAR care pathway has been successfully embedded into NHS care at one hospital. A toolkit and online clinician training package supports wider implementation.

Cite this article: *Bone Jt Open* 2025;6(6):644–650.

## Introduction

Translation of research evidence into clinical practice can be challenging. There is a gap between research and practice that presents difficulties in translating research findings into a form that is implementable in clinical practice, especially in relation to studies involving complex interventions.^1^ Individual clinicians can make changes in their practice based on evidence for treatments such as prescribing new or ‘off-label’ drugs, while implementing complex interventions may require wider multispecialty buy-in and hospital manager support.^2,3^ Research suggests that change can be difficult: barriers to the translation of evidence into practice include organizational cultures not being cooperative or supportive, unmotivated healthcare professionals, deficiencies in continuing education preventing them from developing the skills to appraise research, and insufficient trust and collaboration between researchers and policymakers.^4^ Effective translation of research findings into clinical practice requires dedicated time and resources. Although surgical trials can be challenging, there have been high-quality trials in orthopaedics; the challenge is then translating these findings into practice.^5-7^

Over 100,000 knee arthroplasties are performed every year in the UK.^8,9^ Around one in five patients will have ongoing pain three months after total knee arthroplasty (TKA).^10,11^ Prior to the Support and Treatment After Replacement (STAR) care pathway, evidence-based treatment options have not been available.^10,12,13^ The STAR care pathway is a complex intervention which has been evaluated in a multicentre randomized controlled trial (RCT) and found to be a clinically important and cost-effective treatment for improving outcomes for patients with pain three months after TKA.^14^ This personalized intervention may prevent patients transitioning from postoperative pain at three months to chronic pain in the longer term.^15^

We describe how research findings were translated and implemented into NHS practice at a single centre, which was the lead trial centre, and the further work undertaken to support wider implementation, including development of a comprehensive online training package and toolkit.

## Methods

### The STAR care pathway

The STAR care pathway is a complex intervention that was developed, tested in a multicentre RCT, and proven to be clinically important and cost-effective.^14,16-19^ The STAR care pathway involves screening to identify patients in pain three months after TKA (defined as a score of ≤ 14 on the Oxford Knee Score (OKS) pain subscale).^20-23^ Patients with pain at three months are then invited to a one-hour assessment with a trained healthcare professional to identify the potential cause(s) of the pain and are referred to existing services for appropriate treatment, with telephone follow-up over one year following the assessment. In the RCT, participants randomized to the STAR care pathway had better pain outcomes at one year after randomization, compared with those who had usual care alone. The mean between group difference in the Brief Pain Inventory (BPI)^22^ severity score was −0.65 (95% CI −1·17 to −0·13; p = 0.014) and BPI interference score was −0.68 (−1.29 to −0.08; p = 0.026).^14,24^ The BPI is scored using a numeric rating scale of 0 to 10, with a minimal clinically important difference for the interference and severity scales of 1 point. The cost-effectiveness analysis found that the STAR care pathway offers an NHS cost saving of £724 per treated patient during the period of three to 15 months after TKA surgery, primarily due to decreased inpatient admissions and shorter length of stay. Assuming the trial centres are representative of all NHS units, implementation of the STAR care pathway into usual NHS care would translate to a potential for improved pain and recovery for 20,000 UK patients and NHS cost savings of over £14 million per year, including a yearly reduction of 12,000 inpatient admissions.

### Usual care pilot

A pilot was proposed to adapt trial processes for NHS usual care and provide proof of concept of translation into practice. As the project comprised implementation of evidence into usual NHS care rather than research, research ethics approval was not required. Trial findings and a business case for a usual care pilot were presented to orthopaedic managers at North Bristol NHS Trust (NBT). NBT was the sponsor and lead centre for the STAR trial, which provided the benefit of an engaged consultant lead (NH) and research team with trial knowledge and experience.

The initial business case to orthopaedic managers was based on the benefits of cost savings and improved patient outcomes. At the time of presentation (October 2021), issues dominating the NHS included rising hospital admissions due to COVID-19.^25^ Fewer TKAs were being performed due to infection control restrictions and bed availability, and the NHS was focused on managing upcoming winter pressures with an emphasis on inpatient stays, length of stay (LOS), and bed availability.^26^

The research team emphasized that cost savings were due to decreased inpatient readmissions and LOS after surgery, between three and 15 months after surgery. The research lead (WB) presented additional trial data: inpatient stays (readmissions) in the intervention group were half compared with usual care (mean 0.13 vs 0.25 days per patient), and LOS for those with admissions in the intervention group were 5.8 days shorter than usual care (mean 5.6 vs 11.4 days). Cost saving in the intervention group was primarily due to reduced inpatient activity; the incremental net monetary benefit at £20,000 per quality-adjusted life-year threshold was £1,256 (95% CI 164 to 2,348), with a 98.79% probability of cost-effectiveness compared with usual care.^14^ This was presented with TKA procedure data, including estimated cost savings, number of reduced admissions, and reduction in number of inpatient days. Following review of these data, orthopaedic managers agreed to support the pilot if funding could be obtained.

The orthopaedic general manager (TW) and lead researcher (WB) applied for NHS elective recovery funding. This involved preparation and submission of a detailed business case to NBT senior management comprising costs of delivery, overview of the pilot plan, benefits to elective recovery and urgent and emergency care, risk mitigation strategies, required workforce for delivery, and a detailed outline of impact by month, including reductions in nonelective admissions and LOS.

NHS elective recovery funding was granted by NBT in November 2021, covering delivery costs to the end of the NHS financial year (31 March 2022). Time required for delivery was based on an estimated 30 TKA procedures per month yielding six new care pathway patients per month, requiring five clerical hours, and up to eight practitioner hours per month. Funding was subject to gaining support from all NBT consultant surgeons performing TKAs.

Before the pilot, the lead consultant (NH) and lead researcher (WB) engaged with all consultant knee surgeons, presenting trial findings and pilot processes. Their agreement to the pilot was provided during the December 2021 consultant meeting. The pilot ran from January through March 2022.

The STAR care pathway was delivered by the same two NHS clinicians who underwent training and delivered the intervention during the trial. Screening processes, administration, and facilitation of clinics were undertaken by members of the research team who were responsible for these activities during the trial. Electronic screening methods were proposed by hospital management. A discussion took place regarding the preferences of trial participants, including the decreased uptake of electronic outcome completion. Based on this, it was agreed that the postal approach used in the trial would also be used in the pilot.

### Trial screening document adaptation

Trial documentation required adaptation for use in usual care. Screening materials were adapted for use in the NHS using the capability, opportunity, motivation and behaviour (COM-B) model^27^ for behaviour change and evidence-based approaches to increase the return of postal questionnaires.^28^

The trial postal screening pack included a covering letter, participant information leaflet, consent form, contact details form, two-page OKS, and questions about follow-up appointments, including whether they would like to receive study results and a free-text comments box. These documents were unsuitable for usual care because of references to the trial, randomization, and equipoise. Documents were updated to remove references to the trial, address patient feedback, and describe the new evidence from the STAR trial.

It is well understood that people with pain after TKA can feel abandoned.^29^ Many have a sense of futility, believing that nothing can be done for their pain, and do not seek treatment after their operation.^30^ They are often unprepared for the severity and impact of the pain on their lives and experience low points.^31^ Such people could benefit from information to help them understand that treatment is available.

Previously unpublished feedback from patients and trial participants that might affect completing and returning the questionnaires included: ‘you fill out the forms, but no one ever bothers to look at them’, ‘nothing more can be done’, ‘what can they do?’, ‘you can’t talk to an actual person’, and ‘I don’t want to be a bother’.

The COM-B model for behaviour change was used to create statements addressing new evidence and patient feedback from the STAR trial, with a target behaviour of completing and returning the screening questionnaire.^27^ Psychological capability and reflective motivation behaviours were addressed through education and incentivization behaviour-change techniques, by explaining in the letter that there are treatments to help, and that if their answers show that they need help they will be contacted within two weeks.

The screening pack for the pilot was limited to two pages: the covering letter and OKS, printed double-sided. This was to limit the burden on patients and increase the return rate, as shorter questionnaires yield higher responses.^28^ A template of the covering letter is provided in the Supplementary Material.

### Trial site pilot capacity assessment

NHS hospitals (n = 7) that took part in the trial were contacted by email to understand their capacity to pilot STAR. Information was provided on trial findings and the Bristol pilot, including justification and resources required for delivery.

## Results

### Usual care pilot

All patients who had a TKA for osteoarthritis at NBT were sent the adapted invitation letter and an OKS screening questionnaire by post. Screening response rate for the pilot was 89% (n = 16/18). Overall, two of the 16 patients returned an eligible score (≤ 14 on the OKS pain subscale) and were sent an appointment. Both attended clinic and engaged with treatment.

Extended scope practitioners (ESPs) delivering the intervention used the trial’s training manual and offered feedback on adaptations for usual care.

At the end of the pilot, NHS Trust management agreed to permanently continue and fund the delivery of STAR in usual care. Clinics and follow-up continued with the same trained ESPs, funded by the Trust’s clinical directorate. Facilitation and administration were taken on by NBT staff funded by the Trust, including an administrator and specialist nurse, who were provided with a training and support handover by the research team.

In the year following the pilot (April 2022 to March 2023), 81.2% (n = 251 of 309) of TKA patients responded to postal screening at three months postoperative, and 20% (n = 49 of 251) were eligible to receive the care pathway.^14,23^

### Trial site pilot capacity assessment

Staff from all seven trial sites responded and indicated that a pilot would not be possible due to COVID-19 pressures at the time. Four sites also identified the lack of availability of STAR trained ESPs as an issue. The trained clinicians who delivered the trial intervention were no longer at the Trust, had changed roles, or did not have the capacity to take on additional sessions. This presented a barrier to a usual care pilot and future implementation.

### Training package

Trial training was delivered in a face-to-face setting with a consultant knee surgeon involved in the intervention development (NH). A training manual and ongoing support from the research team was provided. Reproducing this face-to-face training on a national scale was not feasible. To support wider implementation, the trial manual and training sessions were adapted for online delivery in partnership with Health Education England (HEE). This process is described below.

Training package development included consideration of appropriate platforms to deliver training to NHS staff, use of new evidence to support the training, and adaptation of the in-person trial training sessions and instruction manual into a format suitable for national access.

A working group was formed to lead and facilitate the adaptation of the trial training and manual for wider NHS use. Group membership comprised two health services researchers (WB, VW), one orthopaedic surgeon closely involved in the intervention development, trial, and follow-up study (NH), and two additional orthopaedic surgeons not involved in the trial: one from the lead site (MRW) and one from an external hospital (see Acknowledgements). The group was supported by a research administrator involved in trial delivery. The group met regularly to discuss the format, content, modality, and assessment of the training package, and to review and advise on the adaptation of the training manual and training package content. Information was used from the process evaluation conducted during the STAR trial,^32^ and advice was regularly sought from the team delivering the STAR care pathway in usual care, which included two ESP physiotherapists and one specialist nurse (see Acknowledgements).

The working group reviewed the trial intervention training manual and agreed that trial-specific content should be removed and replaced with language appropriate for usual care, and that sections should be added describing new evidence from STAR and updated processes developed during the pilot.

Recommendations for delivery were proposed to provide guidance to NHS Trusts less familiar with the STAR care pathway (e.g. not involved in trial delivery). Provision of these recommendations was agreed to be central to facilitate successful delivery and ensure patient safety and equivalence in standard and quality. The recommendations were reviewed by NBT staff delivering STAR and members of the wider research team involved in trial delivery. Feedback was used to refine the four key elements (screening, assessment, referral, and follow-up) and their definitions.

The manual was reviewed for trial content and adapted to remove references to randomization, equipoise, and trial procedures. The ‘trial overview’ section was removed and replaced with a STAR care pathway overview, including benefits, recommendations for successful delivery, and evidence on patient beliefs and characteristics. Detail was added describing the screening process and practicalities of setting up and facilitating clinics.

The group considered online learning options and identified the HEE NHS Learning Hub as the most suitable as it provides free access for NHS staff.^33^ The lead researcher (WB) engaged with HEE and obtained information about the requirements, costs, and processes involved in the development of e-learning packages. Information was reviewed by the working group, who considered how best to adapt the training to this online format.

The group agreed that a short overview module of 15 to 20 minutes should be developed to explain STAR. This would include the evidence, patient experience, benefits of delivery, and a summary of how the care pathway works. This module would provide details of the screening processes and practicalities of delivering clinics and follow-up. The target audience would include hospital staff involved in the care of TKA patients as well as general practitioners (GPs), hospital managers, and administrative staff.

A more comprehensive training session of up to two hours should cover the delivery of the assessment clinic, referrals, and follow-up in detail. This would comprise the content of the training manual and include videos and photographs of each aspect of the assessment process, including the history, assessment of patient-reported outcome measures (PROMs), physical examination, and allocation of referral(s). Each element was broken down into a long list. The photograph, radiograph, video, or presentation required to demonstrate each assessment was agreed and documented. Agreement was made that a diverse range of presenters should be selected for each example and videos should be kept as short as possible, not exceeding five minutes.

The NBT Clinical Photography department were engaged to create photographs and videos of patients attending clinic. A Patient and Public Involvement group member with experience of TKA volunteered to be videorecorded. The training package was explained in detail to each patient before recording and consent was provided for the use of photographs, videos, and radiographs in teaching, presentation, and publication. Three clinicians involved in delivering STAR volunteered and provided consent to be photographed and videorecorded.

PowerPoint presentations were developed and narrated by members of the research team, describing the content and purpose of PROMs collected before the clinic assessment, including scoring criteria, examples, and referral considerations.

Throughout this process, the working group continued in partnership with HEE to develop and launch the training package, reviewing, testing, and refining each iteration as it was developed. The online training course and toolkit is now available free of charge for NHS staff on the NHS Learning Hub, including overview and clinical training modules, and access to the resources required for delivery.^34,35^

After release of the online training package, two NHS Trusts have initiated implementation of the STAR care pathway. In addition, the Australian National Health and Medical Research Council have granted funding to researchers at the University of Sydney and the University of New South Wales adapt and evaluate the STAR care pathway for use in their healthcare system, primarily via telehealth to enable scalability and accessibility across Australia.^36,37^

## Discussion

The STAR care pathway is a complex intervention that has been successfully implemented into usual care at one NHS hospital. A comprehensive online training package and toolkit is now available to support wider implementation within the NHS.

### Strengths and limitations

This work was focused on the implementation of care in the NHS and may have limited applicability in other healthcare settings. In any setting, appropriate staff and funding are essential to implement the care pathway and need to be in place prior to realizing any cost savings. These savings are accumulated within the secondary care hospital setting, including appointments with physiotherapists, pain services, and surgeons; however, there may be resource implications in other settings, such as referrals for treatment to primary care or community physiotherapy.

Following implementation at one hospital, implementation has been initiated in two further NHS hospitals. In addition, work is being undertaken to alter the care pathway for use in the Australian healthcare system, demonstrating adaptability in other settings.

### Usual care pilot and permanent delivery

A pilot of the STAR care pathway in usual care was delivered successfully under the constrained circumstances of the COVID-19 pandemic. Now permanently part of usual care, patients at NBT with pain after TKA now have access to this care pathway.

This study found that translation into practice was aided by several factors, including the Trust having acted as sponsor of the STAR trial, a motivated research team who were based at the local hospital site to facilitate the pilot, an engaged consultant lead, and the support of dedicated nursing, ESP, and administrative staff funded by the Trust clinical directorate. These staff may not be available at smaller hospitals.

Barriers to delivery include identifying and training the appropriate staff for delivery, and acquiring the upfront costs to employ staff prior to realizing cost benefits from delivery of the care pathway.

### Adaptation of documents

The adaptation of trial screening documents for usual care produced a high response rate, and is an example of the successful use of evidence-based methods to increase responses. Patients who believe that nothing can be done and do not seek treatment for pain after surgery benefit from information that treatment is available. This method utilizes postal screening, which was the preferred method for this demographic during the trial. However, many Trusts are moving towards electronic PROM collection and may prefer to use an electronic format. The issue of digital exclusion should be considered, as Office for National Statistics data indicate that 38.8% of UK adults aged 75 years and over have never used the internet.^38^

### Training package

The online training package and toolkit enables NHS Trusts to overcome the barrier of not having appropriate trained staff available. The overview module offers information to NHS staff who may not be familiar with STAR, explaining the treatment and benefits. This may be of particular interest to GPs, hospital managers, and administrators, NHS staff involved in the care of TKA patients, and staff who intend to deliver STAR and would like an overview before working through the longer clinical training module.

Clear recommendations for delivery are outlined to help Trusts achieve the clinical and cost benefits of the STAR care pathway by ensuring the appropriate staff and supporting arrangements are in place. Recommendations about care pathway delivery are provided for the purposes of safety as well as efficacy. However, Trusts may choose to adapt the care pathway to suit local needs. It is important to note that if the care pathway is modified, it may not realize the same benefits. The evidence base for the care pathway relates to delivery between three and four months postoperatively and we do not have evidence for effectiveness after this timepoint, when patients may have transitioned from pain three months after surgery to chronic pain.^15^

In addition to appropriate staffing, implementation will require buy-in from consultant knee surgeons and NHS managers. This may be best led by an engaged consultant who is familiar with STAR and has completed the training package. The most substantial challenge to wider implementation may be the need for encouraging consultant knee surgeons to change practice and enabling managers to support this change.

In conclusion, the STAR care pathway has been successfully implemented in one NHS hospital. Delivery of an online training package and toolkit addresses barriers to implementation on a national scale. Wider implementation is needed to provide evidence-based care to the one in five patients who will report chronic pain three months after TKA.

**Take home message**

- The Support and Treatment After Replacement (STAR) care pathway is a clinically important and cost-effective treatment for the one in five patients who report chronic pain three months after total knee arthroplasty, and this has been adapted from the trial to be delivered in usual care.

- An online training package and toolkit is available to enable training of staff to deliver the STAR care pathway.

## Data Availability

The data sets generated during the STAR trial are available in the University of Bristol Research data Repository (https://data.bris.ac.uk/data/). Access to the data is restricted to ensure that data are only made available to bona fide researchers for ethically approved research projects, on the understanding that confidentiality will be maintained and after a data access agreement has been signed by an institutional signatory.
